# Correction

**Published:** 1871-01

**Authors:** 


CORRECTION.—Two of the abridged articles in our last number failed to get to the author for proof reading, and, as a result, they present several ridiculous errors.

In the article, "*Apomorphia*," this name is put *Opomorphia;* "in its clinical reaction," should be in its *chemical* reaction; "cholera," should be *chorea.*

In the article on, "*A peculiar inflammation of the lower lip*," the types make us say, "undiscovered," for *undescribed*; "mobular masses," for *nodular* masses; and, "permuculous," for *parenchymous.*

